# Social and emotion dimensional organizations in the abstract semantic space: the neuropsychological evidence

**DOI:** 10.1038/s41598-021-02824-9

**Published:** 2021-12-07

**Authors:** Xiaosha Wang, Guochao Li, Gang Zhao, Yunqian Li, Bijun Wang, Ching-Po Lin, Xinrui Liu, Yanchao Bi

**Affiliations:** 1 State Key Laboratory of Cognitive Neuroscience and Learning and IDG/McGovern Institute for Brain Research, Beijing Normal University, Beijing, China; 2 Beijing Key Laboratory of Brain Imaging and Connectomics, Beijing Normal University, Beijing, China; 3 Department of Neurosurgery, First Hospital of Jilin University, Changchun, China; 4 Institute of Neuroscience, National Yang-Ming University, Taipei, Taiwan; 5 Institute of Science and Technology for Brain-Inspired Intelligence, Fudan University, Shanghai, China; 6 Chinese Institute for Brain Research, Beijing, China

**Keywords:** Language, Long-term memory

## Abstract

An essential aspect of human cognition is supported by a rich reservoir of abstract concepts without tangible external referents (e.g., “honor”, “relationship”, “direction”). While decades of research showed that the neural organization of conceptual knowledge referring to concrete words respects domains of evolutionary salience and sensorimotor attributes, the organization principles of abstract word meanings are poorly understood. Here, we provide neuropsychological evidence for a domain (sociality) and attribute (emotion) structure in abstract word processing. Testing 34 brain-damaged patients on a word-semantic judgment task, we observed double dissociations between social and nonsocial words and a single dissociation of sparing of emotional (relative to non-emotional) words. The lesion profiles of patients with specific dissociations suggest potential neural correlates positively or negatively associated with each dimension. These results unravel a general domain-attribute architecture of word meanings and highlight the roles of the social domain and the emotional attribute in the non-object semantic space.

## Introduction

How does the human brain store the meanings of abstract words with no tangible referents (e.g., “honor”, “relationship”, “technique”)? Behavioral, neuropsychological, and neuroimaging studies have shown that abstract words involve different cognitive and neural processes than words referring to concrete objects^1–6^ (e.g., “chair”, “banana”). For object concepts, after the seminal neuropsychological work reporting category-specific semantic deficits^7,8^, mounting neuropsychological and neuroimaging evidence has revealed that object semantics is organized at least by domains of evolutionary salience (e.g., animals, plants, artifacts^9^) and attributes that associate with certain sensory/motor modalities in the brain (e.g., color, manipulation^10–12^), despite debates about the postulation of a domain-general semantic integration hub in anterior temporal lobe^13,14^. By contrast, the internal structure of the abstract semantic space is extremely elusive. Given the lack of specific external referents, one hypothesis is that abstract word meanings are represented in fundamentally different ways from concrete words and rely on associations possibly derived from language^1,15,16^.

Another compelling, not mutually exclusive, hypothesis is that the domain-attribute structure for concrete object knowledge is (at least partly) generalizable to abstract word meanings with specific parameters (i.e., domains and attributes) being different (see^17–20^ for similar unified frameworks for concrete and abstract semantic representation). For the non-object domains of knowledge salient to humans, one extensively studied domain is social semantic knowledge. Social words—those whose meanings are highly dependent on interpersonal interactions^17,21,22^ (e.g., “relationship”, “honor”)—have been found to evoke stronger activation in a set of brain regions implicated in social cognition (dorsal anterior temporal regions, posterior temporal and parietal regions) compared with nonsocial, similarly abstract, words^21,23–25^ (e.g., “direction”). Impairments of social relative to nonsocial abstract words have been reported in patients with frontotemporal lobar degeneration^26^ and healthy subjects with transcranial magnetic stimulation to the right anterior temporal lobe^27^. Turning to the attributes related to abstract knowledge, the role of emotion has been highlighted^12,13,18^. Emotion significantly influences visual word recognition^28,29^, semantic categorization^30^, and abstract word acquisition^31^. Processing emotional (relative to neutral) verbal stimuli elicits brain activity in regions including the amygdala^25,32^, consistent with the notion that a given experiential semantic property engages brain regions responsible for the corresponding experience^12^. These results suggest that the social domain and the emotional attribute might form part of the structure of abstract concepts. However, these two dimensions have often been examined separately (but see^24^) and call for investigation of their unique effects with the other factor being held constant. Importantly, most evidence comes from neuroimaging studies, where caution is required in drawing inferences about the cognitive architecture due to the correlational nature of the paradigms.

One of the most productive approaches to carve the organization structure of human cognition is through the single-case studies in cognitive neuropsychology: careful documentation of functional dissociation and association patterns in individual brain-damaged patients. This method has been highly fruitful in revealing the domain and attribute organization of the semantic space of concrete objects^9–11^. Here, we employed a multiple-single-case approach^33–35^, analyzing 34 individual patients with brain damage to examine whether brain damage may lead to dissociations between social and nonsocial words and between words that are emotionally valenced and those that are not, while holding the other variable constant, on a word-semantic judgment task. If (at least partly) the brain regions showing activation differences (see above) are also necessary to the processing of the corresponding conceptual dimensions, it would be possible to observe cases with brain damage showing disproportionate deficits of social (relative to nonsocial) or emotional (relative to non-emotional) words (i.e., single dissociations). As the nonsocial/non-emotional words are characterized by the absence of sociality/emotion and comprised of heterogeneous word sets, following the practice of object semantics literature^36,37^, the selective deficits of nonsocial/non-emotional words would be termed as selective sparing of social/emotional words. The double dissociations (i.e., selective deficits and sparing of social or emotional words) would suggest the existence of neural correlates relatively selective and necessary to social or emotional semantic knowledge. The patients’ brain lesions were of diverse aetiologies and anatomical locations, which we documented whenever available to infer the potential neural correlates of abstract word comprehension.

## Results

**Figure 1:**
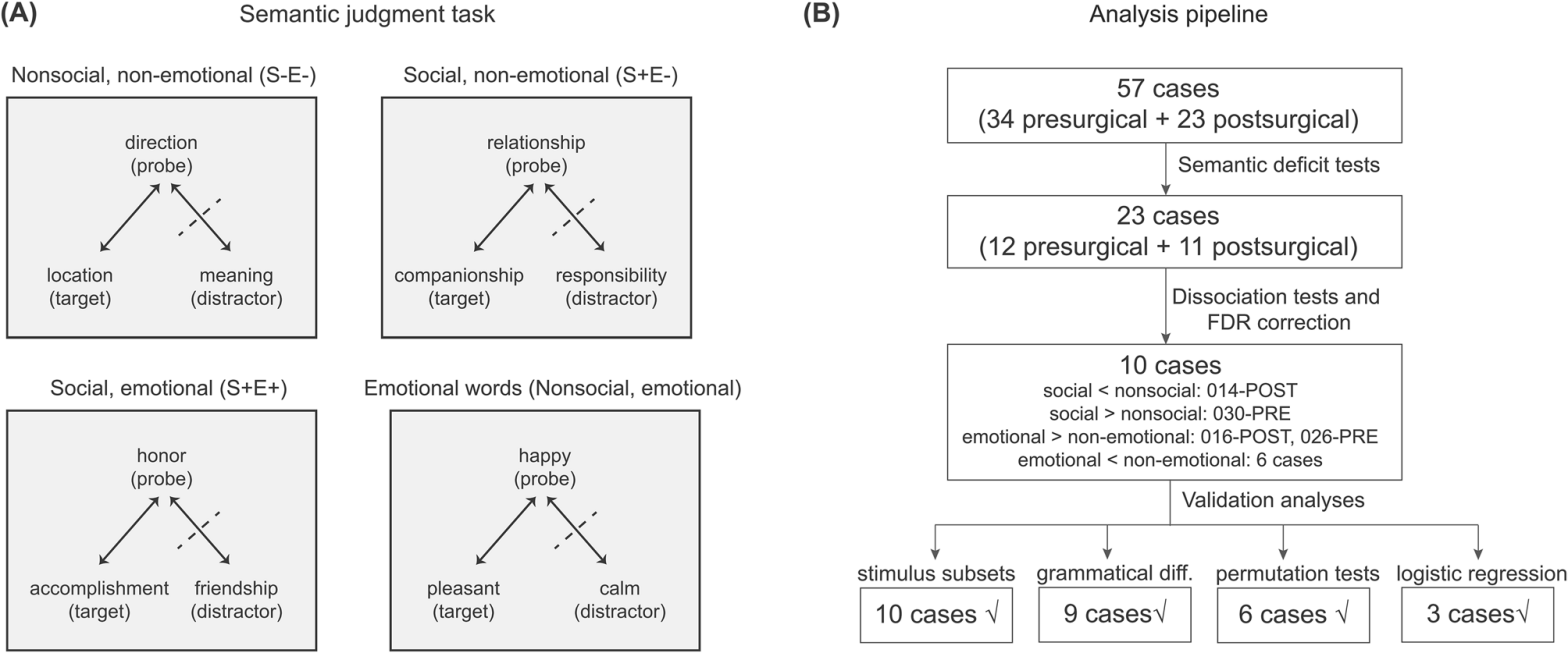
Semantic judgment task (**A**) and scheme of analysis pipeline (**B**) in this study.

To examine whether and how the non-object semantic space breaks down along social and emotional dimensions, we tested patients on a written-word semantic judgment task including four categories differing in social and emotional dimensions (Fig. 1A): nonsocial non-emotional (S−E−, e.g., “direction”), social non-emotional (S+E−, e.g., “relationship”), social emotional (S+E+, e.g., “honor”), and words denoting emotional states (e.g., “happy”). These emotional state words were rated significantly less social than social words (see “Methods” section) and thus could be considered as a nonsocial emotional category (“Emo/S−E+”). Word triplets were constructed for each category and patients were asked to judge which of the two choice words was more semantically related to a probe word. Thirty-four patients completed the task before neurosurgery; 23 of them were also tested shortly after neurosurgery. For each case (34 presurgical and 23 postsurgical), we identified those showing deficits in this task, examined each case’s dissociation across different types of words collected in the same setting (i.e., multiple-single-case approach^33^), and then carried out a series of validation analyses to consolidate those cases with dissociations of interest (see Fig. 1B for the detailed analysis pipeline). Finally, we compared presurgical and postsurgical performances of the same patient to further evaluate the neural separability of different word types in a within-subject fashion. Below each case was labeled with an inclusion number and PRE/POST indicating presurgical or post-surgical tests (e.g., case 001-PRE means the result from the patient (ID, 001), presurgical test).

### Identification and validation of cases showing disproportionate deficits of certain word types

Out of 57 cases (34 presurgical and 23 postsurgical), compared with healthy controls, we first identified 23 cases (12 presurgical and 11 postsurgical) with semantic deficits in at least one category, by comparing each case’s performances with those of healthy controls when controlling for age, years of education, and sex (one-tailed *p*s < 0.05)^38^. For these cases with deficits (Table 1), dissociation tests were then carried out by comparing a case’s accuracies in different conditions [social vs. nonsocial, (S+E+ and S+E−) vs. (S−E− and Emo/S−E+; emotional vs. non-emotional, (S+E+ and Emo/S−E+) vs. (S+E− and S−E−)], which were equivalent to the main effects of sociality or emotion in our approximate 2 (social, nonsocial) × 2 (emotional, non-emotional) design, with reference to the corresponding difference patterns in healthy controls^38^. Dissociation in the neuropsychology literature has been classified as classical (condition A is impaired, condition B is normal, and A differs from B) or strong (both conditions are impaired; A differs from B). Here, the dissociation statistical test we adopted^38^ did not distinguish these two forms of dissociations, as both are informative about whether two conditions have different functional correlates^39^.

**Table 1:** Behavioral performances (proportion correct) of each category in 23 cases with deficits in non-object semantic judgment and validation results for 10 cases with FDR-corrected significant dissociations.

Types of dissociation	Case no	Age, education, sex	Test version	S−E− (%)	S+E− (%)	S+E+ (%)	Emo/S−E+ (%)	Social versus nonsocial: effect size (*p*)	Emotional versus non-emotional: effect size (*p*)	Controlling for grammatical diff.: effect size (*p*)	Permutation (*p*)	Odds ratio (*p*) in logistic regression	
												Raw	Controlling for nuisance factors
Social < nonsocial	014_POST	67, 5, F	Short	88	50	44	88	− ***4.826 (.001)***	− 1.699 (.249)	**− *3.298 (.013)***	***.018***	***0.451 (.033)***	***0.347 (.066)***
Social > nonsocial	030_PRE	50, 5, M	Full	57	88	76	62	***4.667 (.002)***	− 0.779 (.558)	***3.197 (.016)***	***.046***	***1.966 (.015)***	***1.817 (.054)***
Emotional < non-emotional	009_PRE	68, 5, F	Full	86	76	41	69	**− *3.545 (.021)***	**− *5.903 (.0003)***	***5.957 (.00043)***	***.021***	***0.491 (.067)***	0.725 (.565)
Emotional < non-emotional	018_PRE	60, 5, M	Full	79	88	71	62	1.555 (.237)	**− *3.415 (.012)***	***2.677 (.038)***	.103	0.782 (.548)	1.687 (.431)
Emotional < non-emotional	027_PRE	60, 11, F	Full	100	94	65	77	− 1.426 (.247)	**− *5.971 (.00005)***	***5.424 (.00015)***	***.005***	***0.313 (.031)***	0.652 (.602)
Emotional < non-emotional	009_POST	68, 5, F	Short	63	80	44	63	1.138 (.431)	**− *5.085 (.002)***	***3.517 (.011)***	.208	0.967 (.943)	1.237 (.765)
Emotional < non-emotional	018_POST	60, 5, M	Short	75	80	67	63	1.126 (.384)	**− *3.517 (.013)***	1.204 (.328)	.327	0.961 (.936)	1.007 (.992)
Emotional < non-emotional	030_POST	50, 5, M	Full	64	88	53	62	1.372 (.350)	**− *4.017 (.008)***	***5.282 (.00042)***	***.074***	0.690 (.327)	0.708 (.527)
Emotional > non-emotional	026_PRE	45, 8, F	Short	63	30	78	88	− 2.354 (.100)	***6.413 (.0001)***	**− *5.285 (.00057)***	***.024***	***2.407 (.092)***	1.198 (.812)
Emotional > non-emotional	016_POST	41, 16, M	Short	63	70	100	88	1.546 (.222)	***4.497 (.0013)***	**− *2.780 (.028)***	***.050***	***4.147 (.080)***	***34.944 (.024)***
	001_PRE	74, 15, M	Full	93	88	94	77	1.951 (.157)	− 1.920 (.162)				
	022_PRE	55, 5, F	Full	71	71	82	92	− 1.120 (.380)	***2.773 (.034)***				
	033_PRE	61, 3, F	Full	50	53	59	54	1.781 (.342)	0.456 (.797)				
	034_PRE	53, 8, F	Full	64	71	71	62	2.571 (.101)	− 0.814 (.579)				
	037_PRE	46, 5, F	Full	79	76	82	92	− 1.608 (.198)	1.780 (.154)				
	026_POST	45, 8, F	Full	71	71	71	62	1.505 (.319)	− 1.256 (.388)				
	028_POST	38, 9, M	Full	93	82	100	85	− 0.477 (.680)	1.344 (.250)				
	037_POST	46, 5, F	Full	71	71	76	69	0.816 (.566)	0.324 (.815)				
	020_PRE	43, 6, M	Short	63	80	100	75	***3.017 (.019)***	***2.644 (.038)***				
	014_PRE	67, 5, F	Short	38	70	67	88	1.808 (.202)	***2.685 (.070*)**				
	015_POST	49, 15, M	Short	88	100	100	75	***2.671 (.033)***	− 1.955 (.112)				
	027_POST	60, 11, F	Short	75	60	67	100	**− *2.733 (.036)***	1.788 (.178)				
	034_POST	53, 8, F	Short	75	80	78	100	− 0.823 (.481)	1.247 (.296)				

Numbers in bold and italic fonts indicate marginally significant results (*p* < .1). Numbers in bold, italic, and underlined fonts indicate dissociations significant at FDR *q* < .05 across the 23 cases.

*S−E−* nonsocial non-emotional words, *S+E−* social non-emotional words, *S+E+* social emotional words, *Emo/S−E +*, emotional state words with relatively low social ratings, *PRE* examined before surgery, *POST* examined shortly after surgery, *Full* the full-version test, *Short* the short-version test, *F* female, *M* male.

Across 23 cases with semantic deficits, for each contrast, multiple comparisons were corrected at false discovery rate (FDR) *q* = 0.05 (Table 1). Two cases survived the FDR correction for the social versus nonsocial contrast: the case 014-POST was more impaired for social words relative to nonsocial words, and the case 030-PRE was more impaired for nonsocial relative to social words. Eight cases survived the FDR correction for the emotional versus non-emotional contrast: 009-PRE, 018-PRE, 027-PRE, 009-POST, 018-POST, and 030-POST were more impaired for emotional relative to non-emotional words, whereas 026-PRE and 016-POST were more impaired for non-emotional relative to emotional words. We further carried out four validation analyses below to consolidate those dissociations that could not be ascribed to psycholinguistic nuisance variables and were robust to permutation and logistic regression tests.

#### Validation of single-case dissociations in subsets of well-matched stimuli

As mentioned in the “Methods” section, social words were rated to be more concrete than nonsocial words; emotional and non-emotional words were not fully matched on word frequency, concreteness, and visual complexity. To ensure that the dissociations we observed were not driven by these nuisance variables, we controlled for their influence by re-examining patients’ performances in subsets of triplets where these nuisance variables were matched between conditions of interest (see “Methods” section). Table 2 presents patients' performances in these subsets of stimuli. The dissociations remained significant in all the 10 cases identified above (*p*s < 0.039).

**Table 2:** Semantic performance (proportion correct) in the original set and subsets of stimuli matching for psycholinguistic nuisance variables in patients showing FDR-corrected significant dissociations.

Types of dissociation	Case no	Age, education, sex	MMSE	Condition 1	Condition 2	Subsets matched for nuisance variables			Brain lesion locations	Diagnosis and WHO classification
						Condition 1	Condition 2	Effect size (*p*)		
**Social versus nonsocial**				**Social**	**Nonsocial**	**Social**	**Nonsocial**			
Social < nonsocial	014-POST	67, 5, F	n.a.#	9 of 19 (47%)	14 of 16 (88%)	7 of 14 (50%)	13 of 14 (93%)	-4.466 (.001)	L IPL, SPL, PreC, PostC ^&^	Cerebral cysticercosis
Social > nonsocial	030-PRE	50, 5, M	19	28 of 34 (82%)	16 of 27 (59%)	20 of 25 (80%)	15 of 25 (60%)	3.887 (.008)	R MFG, IFG, STG	Meningioma, microcystic, grade I
**Emotional versus non-emotional**				**Emotional**	**Non-emotional**	**Emotional**	**Non-emotional**			
Emotional < non-emotional	009-PRE	68, 5, F	27	16 of 30 (53%)	25 of 31 (81%)	10 of 16 (63%)	14 of 16 (88%)	− 3.785 (.006)	L SPL	Metastatic tumor
	018-PRE	60, 5, M	25	20 of 30 (67%)	26 of 31 (84%)	10 of 16 (63%)	13 of 16 (81%)	− 3.161 (.018)	n.a	Atypical meningioma
	027-PRE	60, 11, F	27	21 of 30 (70%)	30 of 31 (97%)	12 of 16 (75%)	15 of 16 (94%)	− 3.093 (.014)	R SFG	Meningioma, transitional
	009-POST	68, 5, F	n.a	9 of 17 (53%)	13 of 18 (72%)	4 of 8 (50%)	7 of 10 (70%)	− 3.097 (.030)	L SPL, PostC, Precuneus, SMG	Metastatic tumor
	018-POST	60, 5, M	n.a	11 of 17 (65%)	14 of 18 (78%)	4 of 8 (50%)	7 of 10 (70%)	− 3.062 (.029)	n.a	Atypical meningioma
	030-POST	50, 5, M	n.a	17 of 30 (57%)	24 of 31 (77%)	7 of 16 (44%)	13 of 16 (81%)	− 5.531 (.0003)	R MFG, IFG	Meningioma, microcystic, grade I
Emotional > non-emotional	026-PRE	45, 8, F	25	14 of 17 (82%)	8 of 18 (44%)	7 of 8 (88%)	6 of 10 (60%)	2.595 (.039)	L SFG, MFG, IFG	Glioblastoma, grade IV
	016-POST	41, 16, M	n.a.#	16 of 17 (94%)	12 of 18 (67%)	7 of 8 (88%)	5 of 10 (50%)	3.303 (.016)	L IFG, STG, Insula	Anaplastic oligoastrocytoma

*n.a.* not available, *L* left, *R* right, *IPL* inferior parietal lobule, *SPL* superior parietal lobule, *PreC* precentral gyrus, *PostC* postcentral gyrus, *MFG* middle frontal gyrus, *IFG* inferior frontal gyrus, *STG* superior temporal gyrus, *SFG* superior frontal gyrus, *SMG* supramarginal gyrus, *F* female, *M* male.

^#^: 014-POST, presurgical MMSE score = 19; 016-POST, presurgical MMSE score = 28; &, lesion locations in the case’s presurgical MRI scan are shown because her postsurgical MRI scan is not available.

#### Validation of single-case dissociations after controlling for grammatical differences

Our Emo/S−E+ word triplets were mainly composed of adjectives and other categories were nouns. To examine the possibility that the observed dissociations may be driven by such a grammatical difference, we carried out a validation analysis excluding this category and including conditions where grammatical categories were identical (i.e., S+E− vs. S−E− for the social vs. nonsocial dissociation, S+E+ vs. S+E− for the emotional vs. non-emotional dissociation). The dissociations remained significant in 9 cases (*p*s < 0.038, Table 1), which suggests that these dissociation patterns could not be simply attributed to grammatical differences between Emo/S−E+ and other words. The exception case was 018-POST: his disproportionate deficits of emotional relative to non-emotional words did not approach significance (S+E+ vs. S+E−: 67% vs. 80%, effect size = 1.204, *p* = 0.328).

#### Validation of single-case dissociations by permutation tests

Is it possible that the observed dissociations simply reflect random effects of any grouping of the stimuli instead of being meaningful semantic organizations as the social and emotional categories we designed? We tested this possibility using permutation. For each of the 10 cases with significant dissociations along social or emotion dimensions identified above, we randomly permutated category labels 10,000 times and re-calculated accuracy differences between categories to obtain a null distribution of categorical differences while holding the overall impairment severity constant. The observed categorical differences along the social vs. nonsocial dimension for the cases 014-POST and 030-PRE ranked top 175 and 460 out of 10,000 permutated differences, respectively (i.e., *ps* < 0.046). The observed differences along the emotional vs. non-emotional dimension for 4 out of 8 cases (009-PRE, 027-PRE, 026-PRE, and 016-POST) ranked within top 500 (i.e., *p*s < 0.05) and for other 4 cases (all emotional < non-emotional: 018-PRE, 009-POST, 018-POST and 030-POST) ranked above 740 (i.e., *p*s > 0.074, *p* range: 0.074-0.327) out of 10,000 permutated differences (Table 1). This indicates that the social/nonsocial dissociations for 014-POST and 030-PRE and the emotional/non-emotional dissociations for 009-PRE, 027-PRE, 026-PRE, and 016-POST were statistically not by chance.

#### Validation of single-case dissociations by logistic regression tests

We carried out logistic regression analyses to treat social/emotional ratings as continuous measures and to statistically control for a series of psycholinguistic factors. Specifically, for each of the 10 cases showing significant dissociations, using a step-by-step procedure, we first examined the explanatory power of continuous social or emotional ratings to triplet accuracy (Table 1). Significant or marginally significant effects were found in 6 out of 10 cases (*p*s < 0.092), not in the 4 cases (all emotional < non-emotional: 018-PRE, 009-POST, 018-POST, and 030-POST, *p*s > 0.327). These results were consistent with the permutation results above and suggest that dissociations in these 4 cases were not robust enough. We then added 4 nuisance variables (the number of strokes, word frequency, concreteness, semantic distance; for the step-by-step results, see Table S1) and found that social or emotional effects reached or approached significance in 3 cases: 014-POST (social < nonsocial, odds ratio of social ratings = 0.347, Wald z = 3.391, *p* = 0.066), 030-PRE (social > nonsocial, odds ratio of social ratings = 1.817, Wald z = 3.722, *p* = 0.054), and 016-POST (emotional > non-emotional, odds ratio of hedonic valence ratings = 34.944, Wald z = 5.062, *p* = 0.024). Taken together, the logistic regression analysis validated the double dissociations between social and nonsocial words and selective sparing of emotional words and precluded further conclusions about selective deficits of emotional words.

### Details of cases with significant dissociations along social and emotional dimensions

Three cases (2 for social-nonsocial dissociations, 1 with selective sparing of emotional words) survived all of the above-mentioned validations and are considered to be cases showing robust dissociations. In this section, we reported these cases in greater detail.

#### Double dissociations between social and nonsocial words in the brain

**Figure 2:**
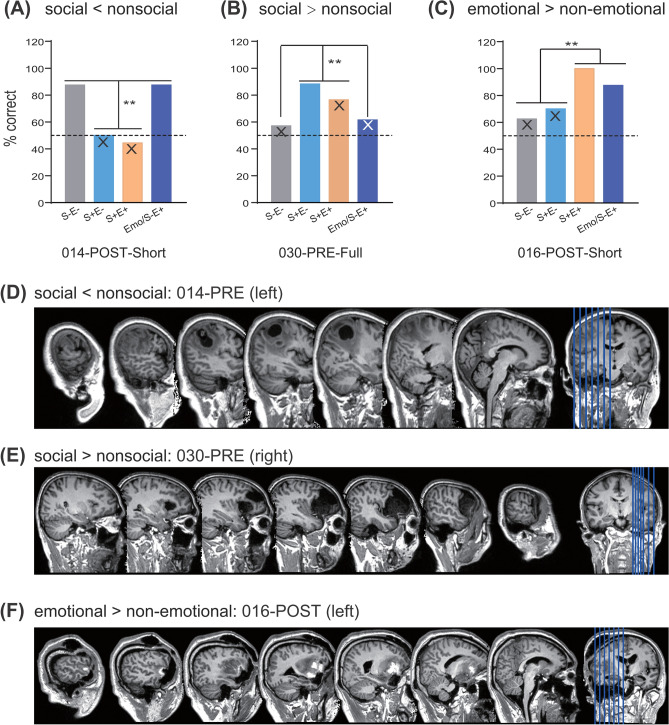
Behavioral performances (**A**–**C**) and lesions (**D**–**F**) of cases showing significant dissociations that survived all the validation analyses. Asterisks indicate significant dissociations, when compared with 28 healthy controls and controlling for three demographic variables (**, *p* < .01, single-case dissociation tests^38^). The X marks indicate significant deficits in a given category compared with 28 healthy controls while controlling for three demographic variables (one-tailed *p* < .05,^38^). The brain slices are visualized using MRIcron^69^. Note that **D** shows the presurgical MRI scan of Patient 014, who did not have available postsurgical MRI scans. S−E−, nonsocial non-emotional words; S+E−, social non-emotional words; S+E+, social emotional words; Emo/S−E+, emotional state words with relatively low social ratings. *PRE* examined before surgery, *POST* examined shortly after surgery, *Full* the full-version test, *Short* the short-version test.

*Impaired social words relative to nonsocial words* was found in 014-POST, with her standardized differences between social and nonsocial words significantly larger than those of controls while controlling for demographic variables (effect size = − 4.83, *p* = 0.0011, Fig. 2A). She was impaired at social words, irrespective of emotional valence (S+E−: correct on 5 out of 10 trials, 50%, effect size = − 4.87, *p* = 0.00016; S+E+, 4 out of 9 trials, 44%, effect size = − 5.20, *p* = 0.00008), but within the normal range for nonsocial words, also irrespective of emotional valence (S−E− and Emo/S−E+, both correct on 7 out of 8 trials (87.5%), effect sizes > − 1.11, *p*s > 0.17). She did not have available postsurgical MRI scans and was impaired in the left parietal lobe according to her presurgical scan (Fig. 2D).

*Relative sparing of social words* was found in 030-PRE (effect size = 4.67, *p* = 0.002, Fig. 2B). Compared with controls, he was severely impaired at two types of nonsocial words (S−E− and Emo/S−E+, correct on 8 out of 13 or 14 trials, 57–62%, effect sizes < − 4.13, *p*s < 0.00069), mildly impaired for S+E+ words (correct on 13 out of 17 trials, 76%, effect size = − 2.35, *p* = 0.026), and within the normal range for S+E− words (correct on 15 out of 17 trials, 88%*,* effect size = − 0.81*, p* = 0.24). The case suffered from a large meningioma in his right frontal lobe (Fig. 2E), which induced the atrophy of the right middle and inferior frontal regions and the suppression of the superior temporal region.

#### Single dissociation between emotional and non-emotional words in the brain

*Relative sparing of emotional words* was found in 016-POST (effect size = 4.50, *p* = 0.0013; Fig. 2C). He was severely impaired at two types of non-emotional words (S−E−, correct on 5 out of 8 trials, 62.5%, effect size = − 3.07, *p* = 0.007; S+E−, correct on 7 out of 10 trials, 70%, effect size = − 2.77, *p* = 0.013), and within normal range at two types of emotional words (accuracy, 88–100%, effect sizes > − 1.01, *p*s > 0.197). MRI scans showed his lesions in the left inferior frontal, insula, and dorsal anterior temporal regions (Fig. 2F). Moreover, the patient’s presurgical performance in non-emotional words was within the normal range (correct on 28 out of 31 trials, 90%, effect size = − 0.32, *p* = 0.395), which indicates that his non-emotional word deficits could not be attributed to premorbid unfamiliarity.

### Within-subject presurgical and postsurgical comparisons: can neurosurgery affect abstract word types in different ways?

Among the 23 patients that were tested both before and after neurosurgery, 13 patients showed semantic deficits in either presurgical or postsurgical test (Table 3). In this section, we compared the pre- and post-surgical performance of each condition in each patient, with reference to healthy controls and controlling for demographic variables using the case-to-case comparison^40^. There were indeed various neurosurgery effects (e.g., general improvement due to surgical removal of huge meningioma, or deficits or improvement in only one condition; no visible general practice effects across patients, paired *t*s < 0.36, *p*s > 0.72). Critically, to have a stringent test for dissociation, we focused on those cases showing the opposite changes in two contrasting conditions (e.g., damage in social words and improvement in nonsocial words). This was done because while there are two sources of variables (practice and surgery) explaining the changes between the pre- and post-tests, cases showing reverse directions of changes cannot be explained without assuming word-category dissociation originating from either or both of these variables (practice and/or surgery). Two such cases were observed: (1) After neurosurgery, the patient 027’s performance improved for emotional words (PRE, 65% (11/17) → POST, 82% (14/17); *p* = 0.047), but significantly worsened for non-emotional words (PRE, 94% (17/18) → POST, 67% (12/18), *p* = 0.006). (2) After neurosurgery, the patient 014’s performance significantly improved for nonsocial words (PRE, 62.5% (10/16) → POST, 87.5% (14/16); *p* = 0.007), but showed a trend to decline for social words (PRE, 68% (13/19) → POST, 47% (9/19); *p* = 0.052). These results suggest neural segregation between social and nonsocial words or between emotional and non-emotional words.

**Table 3:** Presurgical and postsurgical performances (proportion correct) in individual patients.

Neurosurgery effects	Case no	Age, education, sex	Test version	Social		Nonsocial		Emotional		Non-emotional	
				PRE (%)	POST (%)	PRE (%)	POST (%)	PRE (%)	POST (%)	PRE (%)	POST (%)
Emotional ↑, non-emotional ↓	027	60, 11, F	Short	68	63	94	88	***65***	***82***	***94***	***67***
Social ~ ↓, nonsocial ↑	014	67, 5, F	Short	***68***	***47***	***63***	***88***	76	65	56	67
Non-emotional ↑	026	45, 8, F	Short	53	74	75	75	82	76	***44***	***72***
Nonsocial ↓	009	68, 5, F	Short	58	63	***88***	***63***	59	53	83	72
Nonsocial ↓	037	46, 5, F	Full	79	74	***85***	***70***	87	73	77	71
Nonsocial ↑, non-emotional ↑	020	43, 6, M	Short	89	100	***69***	***100***	88	100	***72***	***100***
Nonsocial ↓, non-emotional ↓	016	41, 16, M	Short	95	84	***94***	***75***	100	94	***89***	***67***
All ↑	034	53, 8, F	Short	***53***	***79***	***50***	***88***	***53***	***88***	***50***	***78***
n.s	001	74, 15, M	Short	89	95	88	94	88	100	89	89
n.s	015	49, 15, M	Short	95	100	94	81	88	88	100	94
n.s	018	60, 5, M	Short	74	74	63	69	65	65	72	78
n.s	028	38, 9, M	Full	97	91	96	89	97	93	97	87
n.s	030	50, 5, M	Full	82	71	59	63	70	57	74	77

↑/↓, improvement or impairment at *p* < .05; ~ ↓, impairment at *p* < .1; n.s., *p* > .1, according to case-to-case comparison tests^40^. Numbers in bold and italic fonts indicate conditions with significant or marginally significant neurosurgery effects.

*PRE* examined before surgery, *POST* examined shortly after surgery, *Full* the full-version test, *Short* the short-version test, *F* female, *M* male.

## Discussion

In this study, we investigated the organization structure of abstract word meaning by testing whether brain damage may lead to relative dissociations along the social (domain) and the emotional (attribute) dimensions. Using single-case-series dissociation analyses, we compared semantic judgment performance across non-object word categories varying in social and emotional information in patients with brain damage. We observed double dissociations between social and nonsocial words and a single dissociation of sparing of emotional (relative to non-emotional) words. Below, we discuss the social and emotional results in turn.

### The social semantic knowledge in the human brain

We found that words referring to social activities (e.g., “business”) and entities/properties (e.g., “membership”) could be disproportionately impaired (i.e., case 014-POST) relative to nonsocial words (e.g., “direction”). Such a dissociation could not be explained by emotional differences because the dissociation still holds in emotionally neutral words, unlike previous studies where the social words also tended to be emotionally valenced^26,27^ (e.g., “polite”). The social effect could not be attributed to word frequency, concreteness, or semantic relatedness, either, which have been carefully controlled for. We also observed one case with relative sparing of social words (i.e., 030-PRE). Such a double dissociation provides evidence for the existence of a neural system relatively specific and necessary to social semantic knowledge.

Social knowledge has been broadly and variously defined in the literature, ranging from person memory to social word meanings^41^. Previous studies have reported selective semantic deficits of the knowledge for people^42,43^. Here, our findings of non-people-specific social word meanings add another line of evidence to the social domain in semantic memory. Cognitively, compared with person memory, social word meanings are less associated with episodic memories, personal preferences, or specific perceptual characteristics (e.g., a person’s face or voice)^44,45^. Anatomically, our case (014-POST) was impaired in the left parietal lobe (Fig. 2D and Table 2), which does not overlap with lesions associated with person memory deficits in the left or right anterior temporal lobe^42,43^ and does not cover the right superior anterior temporal lobe that has been proposed to underlie social (emotionally valenced) concept deficits^26,27,34^. The left parietal region has been associated with various loosely defined social tasks^46,47^ and has also been activated stronger by social relative to nonsocial verbal stimuli^21,24,25^. Our findings therefore provide converging lesion evidence for the involvement of this area in social meaning processing. The case with relative sparing of social words was lesioned in the right lateral frontal cortex, which indicates that this area may be not necessary for processing social words.

### The emotional semantic knowledge in the human brain

Our study also examined the role of emotion in abstract word organization with the single-case approach. The emotional words in our study included two types: social emotional words (e.g., “honor”) and emotion words (e.g., “happy”), which have also been referred to as “emotion-laden” words and “emotion-label” words, respectively^48^. Several patient studies have examined emotional word comprehension^49–51^, using emotion-label words only^51^ or grouping “emotion-laden” words in other categories^50^. These studies adopted group-level comparisons in etiologically homogeneous patients with neurodegenerative diseases (e.g., Alzheimer’s disease, semantic variant of primary progressive aphasia) and did not report significant differences between emotion and other types of non-object words. Here, we examined a large sample of patients with relatively focal lesions that are not confined to particular brain regions. While our cases with emotional relative to non-emotional words did not survive logistic regression analyses, we did observe a case with selective sparing of emotional words that was robust across all validation analyses (case 016-POST). The representational separability between emotional and non-emotional words is further supported by the opposite neurosurgical effects on emotional (improvement) and non-emotional (impairment) words in the patient 027.

Emotion has been proposed to be one important dimension along which abstract semantics is organized^12,17,18,32,52^. In the object semantics literature, damage to a particular object attribute knowledge (e.g., color, manipulation) may be relatively independent of other attributes (e.g., shape) or general semantic processing (e.g., object naming)^10,11,33,53–55^. Here we did not explicitly probe words’ emotional knowledge and instead observed relatively selective deficits of words with weaker emotional connotations. This tentatively suggests that emotional attribute is an intrinsic property of the semantic makeup that may protect general semantic processing of emotional words from damage to the brain areas less related to emotional semantic knowledge (e.g., those lesioned in case 016-POST—the left inferior frontal, insula, and dorsal anterior temporal regions). Neuroimaging meta-analysis studies have shown that emotion processing recruits partially overlapping brain regions with those involved in semantic processing (e.g., lateral and medial prefrontal cortex, bilateral posterior middle temporal regions)^4,56–58^. This raises several possibilities for the neural realization of the emotional semantic makeup—such as in areas specifically related to emotional processing, or in (some of) the overlapping areas between emotional and semantic regions, or by the connections between the two neural systems^59^.

### Broader implications and limitations

Semantic knowledge constitutes the basis for various human cognition, and its neural organization is intricately related to broad domains of cognition. The social and emotional dimensions in semantic representation observed in our neuropsychological study and convergent neuroimaging studies are likely driven by the saliency of social and emotional processes in human phylogenetic and ontogenetic development. It has indeed been shown recently that knowledge about emotion modulates emotional facial expression recognition, which has been traditionally thought to be inborn and universal across individuals and cultures^60–62^. The neural basis of such modulation effects of semantic knowledge and perception warrants further investigation.

This study has several limitations. First, our patients were tested in a task of written words with semantic manipulations. Non-semantic, visual word recognition stage of the reading abilities was not independently measured and was controlled for through validation analyses controlling for visual complexity and frequency. A detailed assessment of semantic processing in multiple tasks in future studies could further validate the dissociations we observed. Second, we focused on the dissociation along the social and emotional dimensions (that is, the main effects of sociality and emotion in our approximate 2 × 2 design) and could not adequately address the possibility of the domain-attribute interaction, due to grammatical differences between Emo/S−E+ and other categories. Future studies are also invited to examine whether the social-related emotion (e.g., shyness, envy) and nonsocial-related emotion (e.g., fear of height) may be further segregated in the human brain. Finally, regarding lesion-behavior mapping, it is challenging to rely on individual brain tumor patients to localize cognitive functions due to tumor-induced functional reorganization and a lack of anatomical precision. Studies with a larger sample size and quantitative lesion-behavior mapping methods would be needed for a stronger conclusion of the localization of social or emotional semantic knowledge.

### Conclusion

Using a multi-single-case approach, we report dissociation patterns in individual brain-damaged cases suggesting that abstract word meanings are organized along the social and emotional dimensions. Such a domain- and attribute-based architecture moves beyond the abstract-concrete dichotomy in semantics by suggesting similar organizing principles for concrete object and abstract word meanings. This framework would be strengthened by identifying other domains and attributes relevant to abstract word meanings^17,20^ and an investigation of how this proposed architecture interacts with language experience^1,63–65^.

## Methods

### Participants

A total of 34 patients (mean age 50.1 ± 13.5 years, ranging from 20 to 74 years; education 9.4 ± 3.9 years, ranging from 3 to 16 years; 17 females) participated in the study. Patients were recruited from the Department of Neurosurgery at First Hospital of Jilin University with the following inclusion criteria: no previous brain injury; brain lesions affecting cortical areas; and able to follow task instructions. Patients were excluded if they complained of any visual word recognition problems. Patients were tested shortly after their admission into the hospital and 23 of them were also tested shortly after their neurosurgery (mean: 11.3 ± 5.0 days; ranging from 6 to 25 days). The term “case” is used to refer to the dataset of a patient collected at a single time point. MRI scans (high-resolution T1, contrast-enhanced T1, and T2-weighted images) were available for 24 presurgical and 14 postsurgical cases and were acquired within 0–2 days of the behavioral tests (except for 5 cases, 4–8 days; no dissociations were observed in these cases). The affected brain regions in each patient were identified by a neurologist who was blind to the aims of the study. A total of 28 healthy control subjects (mean age 43.0 ± 12.9 years, ranging from 22 to 65 years; education 10.1 ± 4.1 years, ranging from 3 to 16 years; 19 females) were recruited from patients’ relatives and hospital staff.

All participants were native Chinese speakers and provided informed written consent. The study was approved by the Ethics Committee of First Hospital of Jilin University and was performed in accordance with relevant guidelines and regulations.

### Procedure and stimuli

The task was a written-word triplet semantic judgment, in which one probe word was presented at the top and subjects were asked to decide which of the two choice words (arranged horizontally at the bottom) was more semantically related to the probe (Fig. 1A). Seventy-two triplets (18 per category, see below for details) were administered, which were split into a short-version test (10 triplets per category) and the remaining 32 trials. The short-version test was performed first (in case a patient was unable to finish the full version due to fatigue), where triplets were pseudo-randomly selected so that the four categories were matched and counterbalanced. Triplets were presented pseudo-randomly (not blocked for 4 categories) and the stimulus order was fixed across participants. Target words were presented about 50% of the times in each visual hemifield for each category. For patients with spatial neglect identified by neurologists, word triplets were re-arranged in vertical lines so that patients could perceive them; in our study, only one patient (ID: 001) showed spatial neglect after neurosurgery. The DMDX program^66^ was used to present stimuli and to record responses. A 60-s response deadline was set for each trial. No feedback was provided during the experiment.

Based on the performance of our healthy control subjects, 10 triplets with mean accuracy < 80% were excluded. A S+E+ triplet was also excluded because its triplet-level valence rating was not valenced enough (hedonic valence = 0.47), leaving 61 triplets (Table S2) in the final analysis (the number of triplets in the full (short) version test: S−E−, 14 (8); S+E−, 17 (10); S+E+, 17 (9); Emo/S−E+, 13 (8)). Below triplet-level ratings and psycholinguistic properties (averaged across 3 words in a triplet) were reported (see also Table S3) and compared between conditions.

The four categories of triplets (S−E−, S+E−, Emo/S−E+, and S+E+) were first constructed by authors’ own judgment (BJW and XSW) to ensure 3 words in a triplet from the same category and confirmed based on social and emotional ratings. Ratings were collected from independent groups of healthy college students (native Chinese speakers) via online survey (https://www.wjx.cn/): sociality (how often the meaning of a word involves an interaction between people^17,21^, 1 = never, 7 = always; 27 subjects), emotional valence (1 = negative, 4 = neutral, 7 = positive; 20 subjects), concreteness (1 = very abstract, 7 = very concrete; 20 subjects), and semantic distance between probe and target or distractor words (1 = unrelated, 7 = very close; 18 subjects). Example words receiving high or low ratings were provided in rating instructions and word lists were randomized for each subject in online survey. At the triplet level, the mean sociality ratings (± SD, range) for each category were: S−E−, 3.14 (± 0.42, range = 2.69–4.16); S+E−, 5.61 (± 0.39, range = 4.94–6.17); S+E+, 5.32 (± 0.38, range = 4.54–6.02); Emo/S−E+, 3.55 (± 0.22, range = 3.27–4.06). The mean hedonic valence (distance from neutrality point, 4) ratings for each category were: S−E−, 0.28 (± 0.11, range = 0.15–0.50); S+E−, 0.32 (± 0.16, range = 0.07–0.55); S+E+, 1.60 (± 0.39, range = 0.93–2.07); Emo/S−E+, 1.72 (± 0.28, range = 1.40–2.15). Emo/S−E+ words were further referenced to Chinese emotional state word databases^67,68^, with the majority (33 out of 39) being eligible. Words in one category never appeared in other categories. In the whole task, only two target words (1 S+E+, 1 Emo/S−E+) were used as distractors in other trials.

For the social versus nonsocial contrast, we compared (S+E− and S+E+) versus (S−E− and Emo/S−E+). The two conditions differed significantly in sociality (5.46 ± 0.41 vs. 3.34 ± 0.39, *t*_*59*_ = 20.48, *p* = 1.71 × 10^–28^) and matched on hedonic valence (separately for emotional and non-emotional words, *p*s > 0.58, Tukey’s post hoc test following one-way ANOVA), semantic distance differences between probe-target and probe-distractor word pairs (*t*_*59*_ = − 1.45, *p* = 0.15), the number of strokes (*t*_*59*_ = − 1.71, *p* = 0.092), and log word frequency (*t*_*59*_ = 0.07, *p* = 0.94). Finally, social words were slightly more concrete than nonsocial words (3.78 ± 0.63 vs. 3.35 ± 0.43, *t*_*59*_ = 3.04, *p* = 0.004).

For the emotional versus non-emotional contrast, we compared (S+E+ and Emo/S−E+) versus (S+E− and S−E−). The two conditions differed significantly in hedonic valence (1.65 ± 0.35 vs. 0.30 ± 0.14, *t*_*59*_ = 19.88, *p* = 7.93 × 10^–28^) and matched on semantic distance differences between the probe-target and probe-distractor word pairs (*t*_*59*_ = − 1.12, *p* = 0.27). Sociality was matched in social words (S+E+ vs. S+E−, *p* = 0.12) and was slightly higher in Emo/S−E+ words than in S−E− words (*p* = 0.028) according to Tukey’s post hoc test following one-way ANOVA. The emotional words were less frequent (1.24 ± 0.38 vs. 2.02 ± 0.43, *t*_*59*_ = − 7.34, *p* = 7.21 × 10^–10^), less concrete (3.40 ± 0.58 vs. 3.78 ± 0.53, *t*_*59*_ = − 2.66, *p* = 0.010), and had more strokes (17.23 ± 3.01 vs. 15.65 ± 1.91, *t*_*59*_ = 2.46, *p* = 0.017) than non-emotional words. We further carried out validation analyses to exclude these potential nuisance variables.

All words consisted of two characters and were bisyllabic. All S−E−, S+E−, and S+E+ words were nouns; Emo/S−E+ words in 11 triplets were adjectives and in the remaining 2 triplets were verbs. This grammatical difference between Emo/S−E+ and other categories was later considered in validation analyses.

Participants were tested in a quiet room in the hospital. This non-object semantic triplet judgment task was part of a larger neuropsychological battery focusing on object knowledge (e.g., object picture naming). The whole battery generally took 1–2 test sessions, with each session lasting no more than 1.5 h, including pauses for rest upon request. The non-object semantic task was typically administered at the beginning part of each session.

### Data analysis

#### Single-case dissociation analyses

For all the cases we tested, we first identified the cases showing deficits in either of the four categories by comparing the case’s accuracy with those of healthy controls. This was carried out using the Bayesian test for a Deficit allowing for Covariates (*BTD_cov*), which controlled for 3 demographic variables: age, years of education, and gender^38^. Cases with one-tailed *p* < 0.05 were considered to have (potential) deficits in semantic judgment of non-object words. For each case with deficits, we then examined whether they exhibited dissociations in social vs. nonsocial contrast and emotional versus non-emotional contrast. This was carried out using the Bayesian Standardized Difference Test allowing for Covariates (*BSDT_cov*)^38^, which compared a case’s standardized difference between two types of words with those of healthy controls in the presence of 3 demographic covariates. The two-tailed *p*s of this test across the cases with deficits were then corrected for multiple comparisons at False Discovery Rate (FDR) *q* < 0.05. For patients completing short-version tests, their deficit and dissociation patterns were compared with the short-version performances of healthy controls.

#### Validation analyses for cases with significant dissociations

Four validation analyses were performed for the cases with dissociation patterns that survived the FDR correction.

1. Matching psycholinguistic confounds in subsets of stimuli. To rule out the potential influence of psycholinguistic nuisance variables on dissociation results, we selected subsets of items to match for word frequency, concreteness, number of strokes, semantic relatedness differences between conditions. The subset for the social vs. nonsocial contrast included 25 triplets per condition in the full version and 14 triplets in the short version (matched on psycholinguistic nuisance variables: full version, *p*s > 0.122, short version, *p*s > 0.098). The subset for the emotional versus non-emotional contrast included 16 triplets per condition in the full version and 8 emotional and 10 non-emotional triplets in the short version (matched on psycholinguistic nuisance variables: full version, *p*s > 0.055, short version, *p*s > 0.199). See Table S2 for these subsets of stimuli.
2. Controlling for grammatical differences: Considering that Emo/S−E+ word triplets were mainly composed of adjectives and other categories were nouns, we excluded the Emo/S−E+ category and re-examined the dissociations in other categories. That is, the social dissociation was examined in the S+E− versus S−E− contrast; the emotional dissociation was examined in the S+E+ versus S+E− contrast.
3. Permutation test: Besides comparison with healthy controls, the significance of word type differences was determined using a permutation test. For each case, we randomly permutated category labels 10,000 times to obtain a null distribution of categorical differences, while holding the overall impairment severity constant, and compared the observed differences in condition accuracies with the null distribution to obtain significance levels.
4. Logistic regression: A logistic regression model was built for each case (with constant modeled), in which the social or emotional information could be treated as continuous measures and psycholinguistic nuisance variables could be statistically controlled for. A case’s accuracy (1 = correct; 0 = incorrect) across all the trials s/he completed was predicted first by the continuous social or emotional ratings and then psycholinguistic variables (number of strokes, word frequency, concreteness, and differences in semantic distances between target-probe and target-distractor word pairs) in a step-by-step procedure. The order of nuisance variables was determined by their theoretical importance together with explanatory power of patients’ performances in a priori logistic regression analyses including each variable alone.

#### Within-subject presurgical versus postsurgical comparison

For patients who were tested both and after surgery and exhibited semantic deficits (in either presurgical or postsurgical tests), we compared his/her pre- and post-surgical performance in each condition, using the program *CTC_Vec_Cov*^40^ *(Compare Two Cases allowing for a Vector of Covariates*) to refer a patient’s scores to those of a control sample while controlling for age, years of education, and gender. If patients performed different versions in the presurgical and postsurgical tests, their short-version accuracies were compared. For each word condition (social, nonsocial; emotional, non-emotional) in each patient, presurgical vs. postsurgical differences at two-tailed *p* < 0.05 were considered statistically significant. Critically, to have a stringent test for dissociation, we focused on those cases showing the opposite changes in two contrasting conditions (e.g., damage in social words and improvement in nonsocial words). That is, for social versus nonsocial or emotional versus non-emotional contrasts, we were testing for a conjunction between a significant neurosurgery effect in one condition (e.g., social) and a significant neurosurgery effect in the other condition (e.g., nonsocial) with an opposite direction.

## Supplementary Information

Supplementary Tables.

## Data Availability

The datasets generated and/or analyzed during the current study are available from the corresponding authors on reasonable request.
