# Supplementary material for: Social and emotion dimensional organizations in the abstract semantic space: the neuropsychological evidence
Source: Sci Rep. 2021 Dec 7;11:23572. doi: 10.1038/s41598-021-02824-9 (PMC8651696; doi:10.1038/s41598-021-02824-9)
Supplement: Supplementary file 1 — Supplementary Tables. [file 41598_2021_2824_MOESM1_ESM.pdf]

Supplementary Information for

Social and emotion dimensional organizations in the abstract semantic space: The neuropsychological evidence

Xiaosha Wang, Guochao Li, Gang Zhao, Yunqian Li, Bijun Wang, Ching-Po Lin, Xinrui Liu, Yanchao Bi

This file includes Table S1-S3.

Table S1. The odds ratios (p values) of sociality or emotion in the logistic regression models including the 4 nuisance variables in a step-by-step procedure.

Dissociation types	Case No.	age, education, sex	Test version	soc/emo	soc/emo + number of strokes	soc/emo + 2 nuisance variables (strokes, frequency)	soc/emo + 3 nuisance variables (strokes, frequency, concreteness)	soc/emo + 4 nuisance variables (strokes, frequency, concreteness, semantic distances)
**social < nonsocial**	**014_POST**	67, 5, F	short	***0.451 (.033)***	***0.424 (.032)***	***0.381 (.027)***	**0.376 (.072)**	***0.347 (.066)***
**social > nonsocial**	**030_PRE**	50, 5, M	full	***1.966 (.015)***	***1.904 (.023)***	***1.948 (.022)***	**1.787 (.057)**	***1.817 (.054)***
emotional < non-emotional	009_PRE	68, 5, F	full	***0.491 (.067)***	***0.494 (.080)***	0.747 (.594)	0.719 (.553)	0.725 (.565)
emotional < non-emotional	018_PRE	60, 5, M	full	0.782 (.548)	1.035 (.940)	1.505 (.519)	1.619 (.449)	1.687 (.431)
emotional < non-emotional	027_PRE	60, 11, F	full	***0.313 (.031)***	***0.301 (.029)***	0.794 (.751)	0.651 (.597)	0.652 (.602)
emotional < non-emotional	009_POST	68, 5, F	short	0.967 (.943)	0.816 (.686)	1.274 (.727)	1.268 (.736)	1.237 (.765)
emotional < non-emotional	018_POST	60, 5, M	short	0.961 (.936)	0.930 (.889)	1.023 (.974)	1.065 (.930)	1.007 (.992)
emotional < non-emotional	030_POST	50, 5, M	full	0.690 (.327)	0.677 (.320)	0.676 (.467)	0.711 (.531)	0.708 (.527)
emotional > non-emotional	026_PRE	45, 8, F	short	***2.407 (.092)***	***2.791 (.065)***	1.276 (.736)	1.183 (.822)	1.198 (.812)
**emotional > non-emotional**	**016_POST**	41, 16, M	short	***4.147 (.080)***	***4.137 (.083)***	***8.898 (.042)***	**15.378 (.027)**	***34.944 (.024)***

Note: Number in bold and italic fonts indicates marginally significant results ($p < .1$). PRE, examined before surgery; POST, examined shortly after surgery; Full, the full-version test; Short, the short-version test; F, female; M, male; soc, sociality ratings; emo, emotional ratings (hedonic valence).

Table S2. Word triplets used in this study (Chinese words and English translations, organized in the testing order).

Test No.	Category	Task version	Probe		Target		Distractor		Social subset	Emotion subset	Sociality	Hedonic valence	Semantic distance	Concreteness	Log word frequency	accuracy in healthy controls
1	S+E-	short	价格	price	经济	economy	宗教	religion	1	0	5.63	0.07	3.67	3.73	2.78	100%
2	S-E-	short	技术	technique	科学	science	情况	situation	1	0	3.40	0.50	3.61	3.45	2.98	89%
3	S+E-	short	职业	profession	工作	work	婚姻	marriage	0	0	5.96	0.38	3.56	4.27	2.41	96%
4	S+E+	short	暴力	violence	黑帮	gangster	勾当	dirty deal	0	0	5.48	1.92	2.28	4.02	0.93	96%
5	S+E-	short	地位	status	身价	price of somebody	整体	entirety	1	1	4.94	0.32	2.72	3.02	1.69	82%
6	S+E+	short	人祸	man-made disaster	丧事	beravement	成见	stereotype	1	0	5.39	2.07	2.33	3.98	0.53	100%
7	S+E+	short	文化	culture	艺术	art	假期	vacation	1	1	4.94	1.27	3.22	3.47	2.11	96%
8	S-E-	short	数量	amount	比例	scale	软件	software	1	0	2.85	0.15	2.94	4.38	1.97	89%
9	S+E-	short	角色	character	身份	identity	民情	situation of people	1	1	5.53	0.13	4.28	3.80	1.29	93%
10	S+E+	short	婚礼	wedding	蜜月	honeymoon	功绩	achievement	0	1	6.02	1.82	4.00	4.78	1.09	100%
11	S+E-	short	供需	supply and demand	市场	market	节日	festival	1	1	5.60	0.38	3.22	4.07	1.84	96%
12	Emo/S-E+	short	焦虑	anxious	烦躁	irritable	冷漠	indifferent	1	0	3.78	1.92	3.61	3.05	0.76	100%
13	Emo/S-E+	short	高兴	happy	愉快	pleasant	冷静	calm	1	1	3.33	1.88	4.33	3.22	1.84	93%
14	S+E+	short	亲情	kinship	手足	brothers/sisters	领导	leaders	0	1	5.86	0.93	3.50	4.82	1.51	93%
15	S-E-	short	态度	attitude	观点	opinion	基因	gene	1	1	4.16	0.25	4.50	3.70	1.93	100%
16	Emo/S-E+	short	镇定	composure	冷静	calm	欣喜	rejoice	1	0	3.27	1.40	3.89	2.97	1.08	100%
17	Emo/S-E+	short	伤心	sad	难过	upset	恐惧	fear	1	1	3.47	1.93	3.56	3.05	1.39	96%
18	Emo/S-E+	short	担心	worried	忧虑	concern	生气	angry	1	1	3.68	1.58	3.00	3.05	1.62	100%
19	S-E-	short	真相	truth	事实	reality	方面	aspect	0	1	2.94	0.28	4.22	3.30	1.94	96%
20	S+E+	short	荣誉	honor	成就	accomplishment	友情	friendship	1	1	5.48	1.78	3.83	3.17	1.73	100%
21	S+E-	short	社会	society	团体	group	同胞	fellow citizen	0	0	6.17	0.55	1.78	4.48	2.36	82%
22	Emo/S-E+	short	舒心	comfort	自在	ease	好奇	curious	0	0	3.36	1.45	3.44	2.72	0.96	89%
23	S+E-	short	课程	course	成绩	grade	玩笑	joke	1	1	5.02	0.52	3.22	4.05	1.80	100%
24	S+E-	short	纪律	discipline	制度	system	网络	network	1	0	5.56	0.25	3.44	3.77	2.23	100%
25	S+E-	short	关系	relationship	交情	companionship	责任	responsibility	1	1	5.42	0.55	2.72	2.77	2.19	86%

26	S-E-	short	过程	process	步骤	step	特征	characteristic	1	1	2.72	0.17	3.61	3.52	1.89	82%
27	S+E-	short	收入	income	经济	economy	舆论	public opinion	1	0	5.84	0.50	2.89	3.72	2.66	100%
28	S-E-	short	方向	direction	位置	location	意思	meaning	1	0	2.69	0.17	3.61	3.72	2.09	93%
29	S-E-	short	证据	evidence	结论	conclusion	作用	role	1	1	3.37	0.33	0.50	3.58	1.76	93%
30	S+E+	short	战争	war	暴乱	riot	封建	feudalism	1	1	5.67	2.00	3.39	4.00	1.26	93%
31	S+E+	short	罪行	crime	惨案	horrible crime	诡计	trickery	1	0	4.77	2.07	0.67	3.83	0.75	96%
32	Emo/S-E+	short	生气	angry	愤怒	rage	惊恐	frightened	1	0	3.51	2.05	3.17	3.15	0.85	96%
33	S-E-	short	个性	personality	风格	style	终点	terminal	1	1	3.38	0.45	4.17	3.62	1.29	100%
34	S+E+	short	誓言	oath	诺言	promise	名言	saying	1	0	5.04	0.98	2.67	3.77	0.98	82%
35	Emo/S-E+	short	喜悦	glad	欢乐	delight	欣慰	gratified	1	0	3.67	2.08	1.78	3.07	1.21	86%
36	Emo/S-E+	other	紧张	nervous	不安	uneasy	伤感	sentimental	1	1	3.35	1.48	3.78	3.08	1.40	96%
37	S+E-	other	人情	human relationship	面子	face	待遇	salary	1	1	5.58	0.25	1.78	2.80	1.48	93%
38	S+E+	other	爱情	love	魅力	glamour	假期	vacation	1	1	4.99	1.60	2.39	3.18	1.56	100%
39	S+E+	other	缘分	predestination	机遇	opportunity	前途	future	1	1	4.54	1.17	2.17	2.63	1.45	96%
40	S-E-	other	方法	approach	措施	measures	距离	distance	1	0	2.81	0.23	3.83	4.33	2.22	96%
41	Emo/S-E+	other	激动	thrilled	兴奋	excitement	欣慰	gratified	1	1	3.54	1.53	2.61	3.00	1.63	86%
42	S+E+	other	榜样	role model	英雄	hero	良心	conscience	0	1	5.51	1.42	2.56	3.90	1.76	100%
43	S+E+	other	谣言	rumor	假话	falsehood	丧事	beravement	1	0	5.30	1.87	3.94	4.25	0.99	100%
44	S-E-	other	数学	mathematics	公式	formula	记忆	memory	1	1	2.74	0.20	2.56	3.97	1.30	93%
45	S+E+	other	谎言	lie	骗局	fraud	丑闻	scandal	1	0	5.35	1.77	2.11	3.73	0.89	93%
46	S-E-	other	问题	question	答案	answer	形式	form	1	0	2.81	0.30	1.94	3.63	2.30	100%
47	S-E-	other	动机	motive	目的	purpose	效果	effect	1	1	3.17	0.23	2.94	3.07	1.69	86%
48	S+E-	other	声明	statement	公告	post	义务	duty	1	1	5.30	0.18	3.83	3.52	1.48	96%
49	Emo/S-E+	other	自豪	proud	骄傲	pride	勇敢	brave	1	1	4.06	1.43	2.39	2.72	1.56	86%
50	S+E-	other	协议	agreement	合同	contract	岗位	job	0	0	6.12	0.20	3.22	5.15	2.03	100%
51	S+E-	other	生意	business	商业	commerce	会员	membership	0	1	6.17	0.23	3.06	4.48	1.80	96%
52	S+E-	other	消息	message	通讯	communication	买卖	transaction	1	1	5.43	0.15	2.44	4.05	1.84	96%
53	S+E-	other	世界	world	国际	international	证词	testimony	1	0	5.05	0.55	4.50	3.62	2.30	100%
54	S-E-	other	方式	mode	手段	means	环境	environment	1	0	3.33	0.32	3.83	3.47	2.42	96%

55	S+E-	other	机构	organization	单位	department	新闻	news	0	0	5.98	0.22	3.72	4.55	2.57	89%
56	Emo/S-E+	other	惋惜	regret	遗憾	pity	惊慌	panic	1	0	3.72	1.50	4.06	2.97	0.79	86%
57	Emo/S-E+	other	快乐	joy	开心	cheerful	安详	serene	1	0	3.40	2.15	3.00	3.17	1.18	100%
58	S+E+	other	冷战	cold war	分歧	disagreement	传闻	hearsay	1	1	5.54	1.12	2.00	3.25	1.22	100%
59	S+E+	other	仇恨	hatred	敌意	hostility	封建	feudalism	1	0	5.14	2.02	3.56	2.63	0.81	93%
60	S-E-	other	性格	character	脾气	temper	重点	focus	1	0	3.56	0.40	4.33	3.52	2.01	96%
61	S+E+	other	纠纷	dispute	矛盾	contradiction	敌情	enemy's situation	1	1	5.44	1.38	2.67	3.28	1.51	96%

Table S3. Example stimuli, ratings, and psycholinguistic properties of non-object words in the full version.

Category	Example	Sociality	Hedonic valence	Semantic distance [a]	Concrete-ness	Log word frequency [c]	No. of strokes	accuracy in healthy controls
nonsocial, non-emotional	方向(direction)	3.1±0.4	0.3±0.1	3.3±1.1	3.7±0.4	2.0±0.4	16.3±1.9	94%±8%
social, non-emotional	关系(relationship)	5.6±0.4	0.3±0.2	3.2±0.8	3.9±0.6	2.0±0.4	15.1±1.8	95%±7%
social, emotional	荣誉(honor)	5.3±0.4	1.6±0.4	2.8±0.9	3.7±0.6	1.2±0.4	16.7±1.8	96%±7%
Emotional state (nonsocial, emotional)	高兴(happy)	3.5±0.2	1.7±0.3	3.3±0.7	3.0±0.2 [b]	1.3±0.4	17.9±4.1	93%±7%

Notes:

a. Semantic distance was measured as the differences of semantic relatedness ratings between the probe-target and probe-distractor pairs.

b. The low concreteness of emotional state words is consistent with previous studies (Altarriba et al., 1999).

c. Obtained from (Sun et al., 1997).

References

Altarriba, J., Bauer, L. M., & Benvenuto, C. (1999). Concreteness, context availability, and imageability ratings and word associations for abstract, concrete, and emotion words. *Behavior Research Methods, Instruments, and Computers*, *31*(4), 578–602. https://doi.org/10.3758/BF03200738

Sun, H., Huang, J., Sun, D., Li, D., & Xing, H. (1997). Introduction to language corpus system of modern Chinese study. In M. Y. Hu (Ed.), *Paper collection for the fifth world chinese teaching symposium* (pp. 459–466). Peking University Publishers.
